# A Quantal Response Statistical Equilibrium Model of Induced Technical Change in an Interactive Factor Market: Firm-Level Evidence in the EU Economies

**DOI:** 10.3390/e20030156

**Published:** 2018-02-28

**Authors:** Jangho Yang

**Affiliations:** Department of Economics, The New School for Social Research, 6 E 16th Street, New York, NY 10003, USA; yangj994@newschool.edu; Tel.: +1-347-276-4512

**Keywords:** induced technical change, statistical equilibrium, bounded rationality, cost minimizing behavior, quantal response, factor price, C10, D01, D21, D22, D80, O33

## Abstract

This paper studies the pattern of technical change at the firm level by applying and extending the Quantal Response Statistical Equilibrium model (QRSE). The model assumes that a large number of cost minimizing firms decide whether to adopt a new technology based on the potential rate of cost reduction. The firm in the model is assumed to have a limited capacity to process market signals so there is a positive degree of uncertainty in adopting a new technology. The adoption decision by the firm, in turn, makes an impact on the whole market through changes in the factor-price ratio. The equilibrium distribution of the model is a unimodal probability distribution with four parameters, which is qualitatively different from the Walrasian notion of equilibrium in so far as the state of equilibrium is not a single state but a probability distribution of multiple states. This paper applies Bayesian inference to estimate the unknown parameters of the model using the firm-level data of seven advanced OECD countries over eight years and shows that the mentioned equilibrium distribution from the model can satisfactorily recover the observed pattern of technical change.

## 1. Introduction

**Figure 1:**
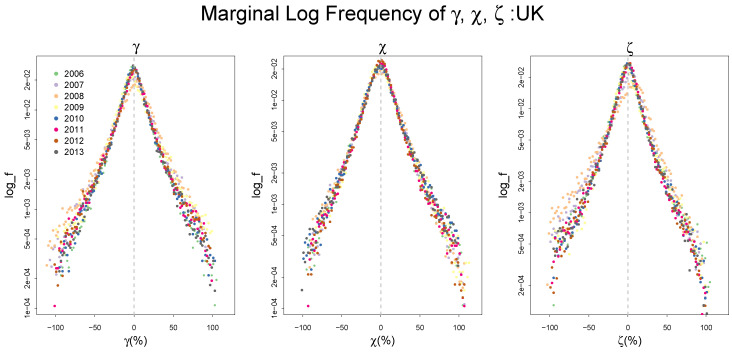
The frequency distribution of the growth rate of labor and capital productivity, γ,χ and the rate of cost reduction ζ of the UK from 2006–2013 with log scale on the vertical axis. The histograms are centered for each year. Both distributions exhibit a peaked tent shape. Data Source: ORIBS-AMADEUS.

One of the most interesting features of the patterns of technical change in the modern economy is that the empirical frequency distributions of the rate of changes in labor productivity, capital productivity, and the realized rate of cost reduction (a negative total factor productivity growth) have a stable shape in many different economies over different time periods. Figure 1 displays the log frequency distribution of these variables for the UK firms from 2006–2013, whose patterns are consistently observed in other countries, as we will see in the following section:

All distributions exhibit the same highly peaked symmetric pattern, yielding a well-behaved tent-shaped distribution. This consistency of the empirical frequency distributions implies that a systematic force in the process of technical change generates what is called in thermodynamics *statistical equilibrium* [1,2]. Statistical equilibrium is defined as the most likely state of the system in the form of a probability distribution. Unlike the conventional notions of equilibrium in economics such as the market-clearing Walrasian equilibrium, whose main theoretical power is to prove the existence of the equilibrium state of the system expressed as the fixed point, statistical equilibrium inherently predicts the central tendency and the fluctuations around it simultaneously. Consequently, it provides a useful framework for analyzing the systemic force underlying the patterns of observed frequency distributions. (There is a wide body of economics literature on statistical equilibrium applications such as [3,4,5,6,7,8,9,10]. For a survey on information theoretic approaches in economics, see [11,12,13,14].)

This paper applies and extends the theoretical framework of the Quantal Response Statistical Equilibrium model (QRSE) recently proposed by [15], and develops a model of two systemic forces that govern the process of firms’ technical progress. The paper also utilizes an entropy constrained model of induced technical change (ITC) as the baseline model of a firm’s behavior in adopting a new technology. The ITC model makes a behavioral assumption that the firm tries to maximize the rate of cost reduction, and therefore, responds with a higher probability to the higher potential cost reduction the new technology would bring about. The firm, however, is assumed to have a limited capacity to process market signals so that there is a positive uncertainty in adopting an optimal technology. On the other hand, the model assumes that the adoption of a new technology by the firm makes an impact on the factor market and adversely changes the factor-price ratio of the firm against the firm’s initial choice.

The equilibrium distribution of this model is a unimodal distribution of the rate of cost reductions with four parameters, qualitatively predicting the observed peaked distribution of the rate of cost reduction. We then utilize a Bayesian inference to recover the four unknown parameters of the model by fitting the derived statistical equilibrium distribution to the observed distribution of a firm’s rate of cost reductions in the seven different advanced EU countries over eight years. The empirical result shows that, during the financial crisis in 2007–2009, the firms became more reluctant to adopt a new technology and needed a higher premium on the potential rate of cost reduction to do so. Also, there was a higher degree of uncertainty in adopting a new technology, so that the economic gains from the technical change were reduced.

The paper consists of four parts. Section 2 provides a description of the data we use in the paper and displays the firm-level frequency distributions of a few technological variables such as the rate of changes in labor productivity, capital productivity, and the realized rate of cost reductions. Section 3 discusses the QRSE model of technical change. The statistical equilibrium of the model will be derived in this section. Section 4 sets up a Bayesian model to estimate the unknown parameters of the statistical equilibrium distribution and recovers the posterior distributions of the rate of cost reductions and the adoption decision. Section 5 discusses further the methodological advantages of the statistical equilibrium approach.

## 2. Patterns of Technical Change

### 2.1. Data

We use the AMADEUS database where OECD firm-level data is available (I am grateful to Columbia University for the access to AMADEUS database.) and extract four variables: wages (W), the number of employees (L), total asset (K), and earnings before interest and taxes (EBIT) for 9 years of observations in 7 different advanced EU countries: the United Kingdom, Germany, France, Italy, Spain, Sweden, and Portugal. The first five countries are the largest EU economies and their combined GPD shares around 70% of the entire EU GDP. Sweden and Portugal are included to see if relatively small sized economies have the same patterns as other larger economies.

Labor productivity *x* and capital productivity ρ are defined as the total value added divided by total labor and total capital invested, respectively. Since the sum of EBIT and wages is the value added of the firm, *x* and ρ in year *t* are calculated as $x_{t}=(W_{t}+{\mathrm{EBIT}}_{t})/L_{t}$ and $\rho_{t}=(W_{t}+{\mathrm{EBIT}}_{t})/K_{t}$. The growth rate of labor and capital productivity is obtained as $\gamma_{t}=\left(x_{t}-x_{t-1}\right)/x_{t-1}$ and $\chi_{t}\;=\;\left(\rho_{t}-\rho_{t-1}\right)/\rho_{t-1}$. For a detailed discussion on the accounting framework, see [16].

Assuming the constancy of the wage and profit rates, the growth rate of cost reduction can be expressed as the average of γ and χ weighted by the unit labor and unit capital costs, $\zeta_{t}=\omega_{t}\gamma_{t}+\pi_{t}\chi_{t}$, where $\omega =W_{t}/(W_{t}+{\mathrm{EBIT}}_{t})$ and $\pi ={\mathrm{EBIT}}_{t}/(W_{t}+{\mathrm{EBIT}}_{t})$, respectively. (ζ can be derived by taking the log derivative of the unit total cost given the wage and profit rate. The total costs *C* is the sum of the total labor and capital cost: C=rK+wL, where r=EBIT/K and w=W/L are the rate of profit and the wage rate. Therefore, $\frac{d(C/Y)}{C/Y}=\omega d\left(x\right)/x+\pi d\left(\rho \right)/\rho =\omega \gamma +\pi \chi$.) It is instructive to note that ζ is mathematically equivalent to the negative total factor productivity growth. We exclude those firms whose value added (W + EBIT) is negative. This effectively sets the lower bound of the growth rates to –100%. For simplicity, we also confine the upper bound of the growth rate to 100%. This is one way of excluding extreme values from the noisy firm-level data. Since the focus of the paper is to analyze some salient patterns of the observed distributions, restricting the data to economically plausible range can be a good first approximation. Around 10% of the data points have been removed after this manipulation. The following is the number of observations for 7 countries for 8 years: the United Kingdom = 188,223, Germany = 86,306, France = 75,894 , Italy = 345,037, Spain = 524,705, Portugal = 181,195, Sweden = 211,237.

### 2.2. Empirical Distributions of γ, χ and, ζ

**Figure 2:**
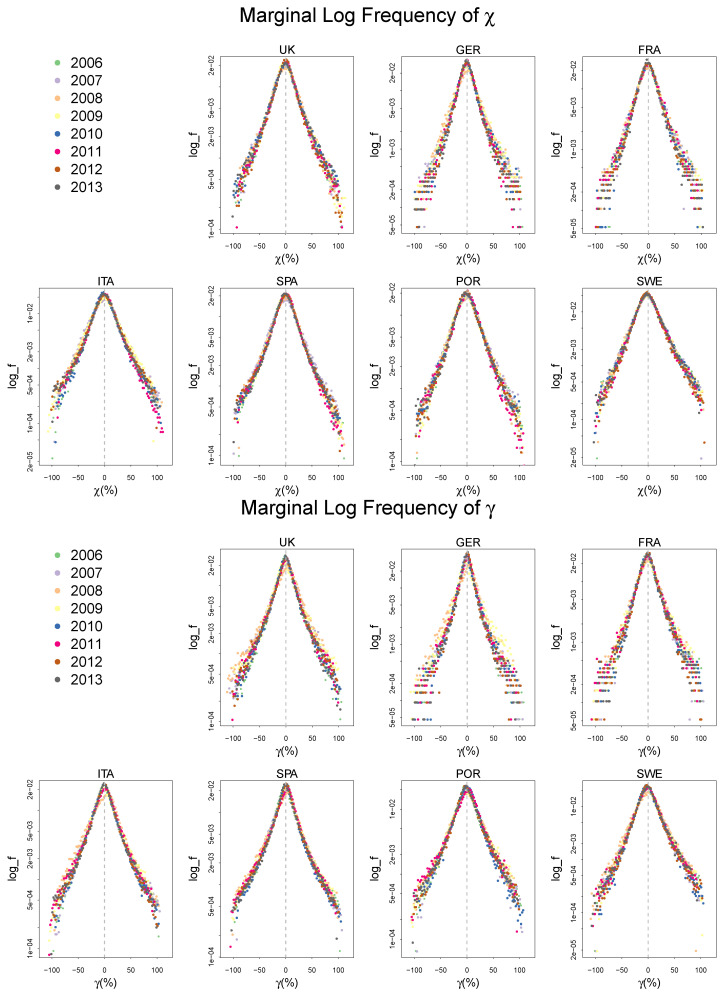
The frequency distribution of the growth rate of labor and capital productivity, γ and χ, with log scale on the vertical axis. The histograms are centered for each country and each year. Both distributions exhibit a peaked tent shape.

We first present the log-frequency distributions of the growth rate of labor and capital productivity, γ and χ in Figure 2. See Appendix A for a summary statistics of the distributions.

**Figure 3:**
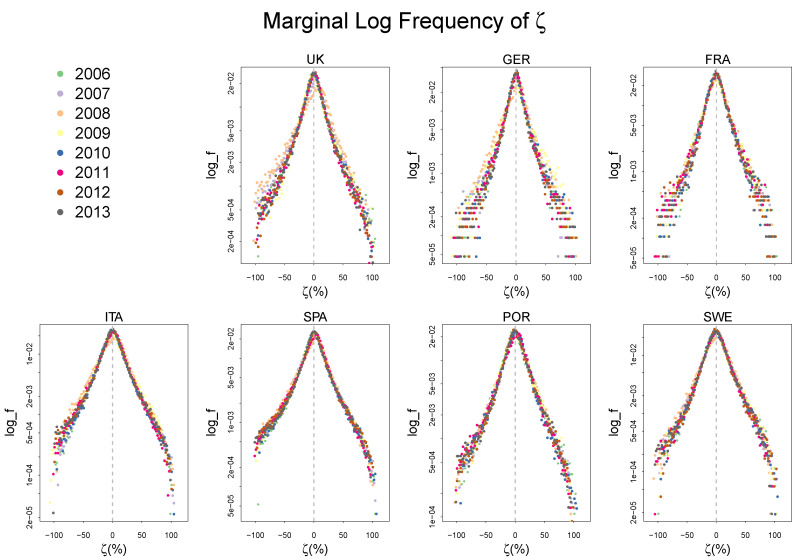
The frequency distribution of the growth rate of cost reduction ζ with log scale on the vertical axis. The histograms are centered for each country and each year. The distribution exhibits a peaked tent shape.

Both γ and χ in all countries in different years show the same patterns as the ones from the UK mentioned in the introduction, exhibiting a fairly stable tent shaped distribution. This strong regularity is also observed in the frequency distribution of ζ as is shown in Figure 3.

The highly peaked symmetric distribution of ζ consistently observed in many different countries over a decade implies that there is a systematic force in the process of technical change that governs the deviation of the rate of cost reduction from its central tendency, which we will explore in the following section.

## 3. A Statistical Equilibrium Model of the ITC

A highly peaked and symmetric pattern of the frequency distributions of γ,χ, and ζ suggest that there is a central tendency in the distribution and structured deviations from it. The observed pattern of technical progress departs from the single-state equilibrium, such as the Walrasian equilibrium, in that the deviation from the central tendency (that is, the mode of the frequency distribution) is persistent and is not properly explained by the normal distribution as is in the case of many stochastic Walrasian models. (The actual fit of the observed data of ζ is better explained by the Laplace distribution $f\left(x|\mu ,b\right)=\frac{1}{2b}\exp\left(-\frac{\left|x-\mu \right|}{b}\right)\;\;$, where μ and *b* are the location and the scale parameter. There are a few notable economic studies deriving and applying the Laplace distribution from the statistical equilibrium perspective. For example, see [17,18] for their studies on the firm profit rates and growth rates.) A more proper concept of equilibrium compatible with the pattern of the observed data is one that predicts the equilibrium as a distribution of different states.

While the notion of equilibrium as a probability distribution is relatively unfamiliar to economists, it has been widely accepted in physics and information theory under the name of *statistical equilibrium* [1,2]. Statistical equilibrium represents the most likely state of the system in the form of a probability distribution p(x), which can be derived by maximizing the entropy of the system, H[p(x)]=−∑p(x)log[p(x)]. (For the discussions on the derivation of different statistical equilibrium from the maximum entropy, see [19,20].) The Walrasian single-state equilibrium is an unattainable special case of this equilibrium when only one state is assigned a positive probability. The statistical equilibrium of most systems in reality does not collapse to a degenerate distribution but assigns a positive probability to multiple states. Therefore, introducing statistical equilibrium to economics suggests that the goal of economic models is to find a non-degenerate probability distribution of the target system that explains the central tendency along with inherent fluctuations around it.

Based on the statistical equilibrium approach, we will introduce one class of model called the QRSE model [15]. Suppose a finite set of outcomes X→R and a finite discrete set of actions $A=\{a_{1},\cdots ,a_{n}\}$. In our example of the firm’s choice of technique, the discrete action variable is a binary set $A=\{a,\bar{a}\}$ consisting of two complementary actions with *a* and $\bar{a}$ indicating the adoption and non-adoption of a new technology, while the set of outcomes consists of the rate of cost reduction expected from the new technology. The key dynamics of this model is that the outcome and action variables interact with each other. First, the quantal response part of the model predicts that the firm decision on the adoption of a new technology is determined in response to the outcome variable, ζ, the degree of potential rate of cost reduction. The impact of the potential cost reduction on the probability of adoption is expressed through the conditional probability of *A* on ζ, p(A|ζ). Second, the model predicts that the rate of cost reduction itself is also affected by the firm’s act of adopting a new technology. The impact of adoption of technology on outcome variables is expressed as p(ζ|A).

Different economic theories of technical change can lead to different specifications of these two-way interactions expressed by p(A|ζ) and p(ζ|A). The following subsections discuss the QSRE model of ITC to specify these interactions and derive the statistical equilibrium distribution of the rate of cost reduction.

### 3.1. The Impact of the Cost Reduction on Adoption of a New Technology

We employ the induced technical change (ITC) model [21,22,23,24,25] as the baseline behavioral model for a choice of technique. In this model, a typical firm maximizes ζ by adopting a new technology constrained by the *innovation possibilities frontier* (IPF), which defines a trade-off between increases in labor and capital productivity. To maximize ζ, the firm adopts a technology that affects γ and χ in response to changes in ω and π. For example, if there is an increase in unit labor cost ω, the firm will be better off introducing a labor-saving technology, which is expressed by increasing γ and decreasing χ.

Following the logic of the ITC model, our model assumes that the probability of the firm adopting a new technique depends on how much cost reduction ζ it can achieve. Therefore, the quantal response function is expressed by the conditional distribution of the adoption decision on the potential rate of cost reduction, p[A|ζ]. The quantal response function has an associated payoff function u[A,ζ] representing the payoff to the typical firm of adopting a new technique. The model assumes that the typical firm maximizes the expected payoff with a mixed strategy of $A=\{a,\bar{a}\}$. This boils down to a simple maximization problem as follows:

(1) $$\begin{array}{cc} \max & \sum_{A}p\left(A|\zeta \right)u\left(A,\zeta \right),\\ s.t & \sum_{A}p\left(A|\zeta \right)=1. \end{array}$$

With no further constraint, the solution to this problem is the Dirac Delta function, choosing to either adopt or not adopt:

(2) $$\begin{array}{c} p\left(A|\zeta \right)=\mathrm{DiracDelta}(A-\hat{A}\left[u,A,\zeta \right]), \end{array}$$

where $\hat{A}\left[u,A,\zeta \right]$ is the choice of adoption or non-adoption that maximizes the payoff. Therefore, the resulting frequency distribution of p(A|ζ) puts unit weight on the payoff-maximizing action and zero weight on the other.

From a statistical equilibrium point of view, however, this result is extremely unlikely to happen because it requires the entropy of the system to be zero. In economics, the zero entropy case can be understood as a *perfect rationality model*, in which the typical firm has a full capacity to process all relevant market signals, resulting in a complete certainty about her decision. In the context of technical change, the perfect rationality model implies that any changes in the input costs will induce an optimal response of technical change so that the potential rate of cost reduction is fully exhausted. Since the zero entropy is not attainable in the real world and remains only as an unrealistic theoretical entity, we need to generalize the model by introducing a positive minimum entropy $H_{min}$. This simple modification is one way to model the *bounded rationality* of the economic agent. Consequently, the model yields a different maximization problem with an entropy constraint as follows:

(3) $$\begin{array}{cc} \max & \sum p(A|\zeta )u(A|\zeta ),\\ s.t & \sum_{a}p\left(A|\zeta \right)=1,\\ & H\left[p\left(A|\zeta \right)\right]=-\sum p\left(A|\zeta \right)\mathrm{Log}\left[p\left(A|\zeta \right)\right]\geq H_{min}. \end{array}$$

A Lagrangian function of this maximization problem is:

$$\begin{array}{ccc} L & = & \sum p\left(A|\zeta \right)u\left(A,\zeta \right)-\mu \left(\sum p(A|\zeta )-1\right)+T\left(\sum p\left(A|\zeta \right)\mathrm{Log}\left[p\left(A|\zeta \right)\right]-H_{min}\right). \end{array}$$

The resulting frequencies of p(A|ζ) at a behavior temperature or “shadow price” *T* is:

(4) $$\begin{array}{c} p\left(A|\zeta \right)=Z{\left(u,T,A\right)}^{-1}e^{\frac{u(A,x)}{T}}=\frac{e^{\frac{u(A,\zeta )}{T}}}{\sum e^{\frac{u(A,\zeta )}{T}}}, \end{array}$$

where the partition function $Z\left(u,T,A\right)=\sum e^{\frac{u(A,\zeta )}{T}}$. The result suggests that the optimal technical change is not a single rate of cost reduction, but a probability distribution of all the possible rates of cost reduction. The behavior temperature *T*, which was originally called *entropy prices* in Foley’s seminal work on the statistical equilibrium approach to economics [4], plays an important economic role because it determines the overall intensity of the payoff of economic actions in the market. As we will see in more detail in the subsequent discussions, higher behavior temperature *T* implies lower intensity of the payoff and thus more uncertainty over the possible economic actions. This form of Quantal Response (QE) model has been used to model bounded rationality in economics. A general survey of QE models can be found in [26,27,28]. One major difference of the QRSE model from the previous models in the QE literature is that our QE model is obtained as an implication of the maximum entropy principle, not a behavioral assumption. (For a seminal work on the QE models from the game-theoretic perspectives, see [29,30,31]. A discussion on the entropy theoretic approach to the bounded rationality can be found in [32]. I appreciate the anonymous referees of this journal for this point.)

Further deriving p(A|ζ) for a binary variable *A*, we have:

$$\begin{array}{ccc} p(A=a|\zeta ) & = & \frac{e^{\frac{u(a,\zeta )}{T}}}{e^{\frac{u(a,\zeta )}{T}}+e^{\frac{u(\bar{a},\zeta )}{T}}}=\frac{1}{1+e^{-\frac{u\left(a,\zeta \right)-u\left(\bar{a},\zeta \right)}{T}}},\\ p(A=\bar{a}|\zeta ) & = & 1-p\left(a|\zeta \right)=\frac{e^{-\frac{u\left(a,\zeta \right)-u\left(\bar{a},\zeta \right)}{T}}}{1+e^{-\frac{u\left(a,\zeta \right)-u\left(\bar{a},\zeta \right)}{T}}}. \end{array}$$

The recovered conditional distribution p(A|ζ) gives the probability of a particular action given observed economic variable ζ. Except for the case when the behavior temperature *T* is zero, the link function is not degenerate and assigns positive probabilities to heterogeneous responses.

The payoff difference $u\left(a,\zeta \right)-u\left(\bar{a},\zeta \right)$ (that is, the difference in payoff between adoption and non-adoption) can be modeled to satisfy the following conditions. First, a higher ζ increases the payoff of adoption while lowering the payoff of non-adoption, and therefore increases the payoff difference. Second, the payoff difference becomes zero so that the firm is indifferent to the adoption of a new technology when ζ is equal to a shift parameter μ that determines the indifference point or the hurdle point of the rate of cost reduction where the probability of adopting a new technology is 50%. This shift parameter μ can be understood as a “premium” on the rate of cost reduction required for the firm to adopt the technology. If μ is high, this implies that the firm needs a higher premium on the rate of cost reduction to adopt the technology with a higher than 50% probability. The simplest linear function that reflects these two constraints is:

(5) $$\begin{array}{c} u\left(a,\zeta \right)-u\left(\bar{a},\zeta \right)=\zeta -\mu . \end{array}$$

Therefore, the impact of the rate of cost reduction on adoption of a new technology is modeled as

(6) $$\begin{array}{ccc} p(A=a|\zeta ) & = & \frac{1}{1+e^{-\frac{\zeta -\mu }{T}}},\\ p(A=\bar{a}|\zeta ) & = & \frac{e^{-\frac{\zeta -\mu }{T}}}{1+e^{-\frac{\zeta -\mu }{T}}}. \end{array}$$

**Figure 4:**
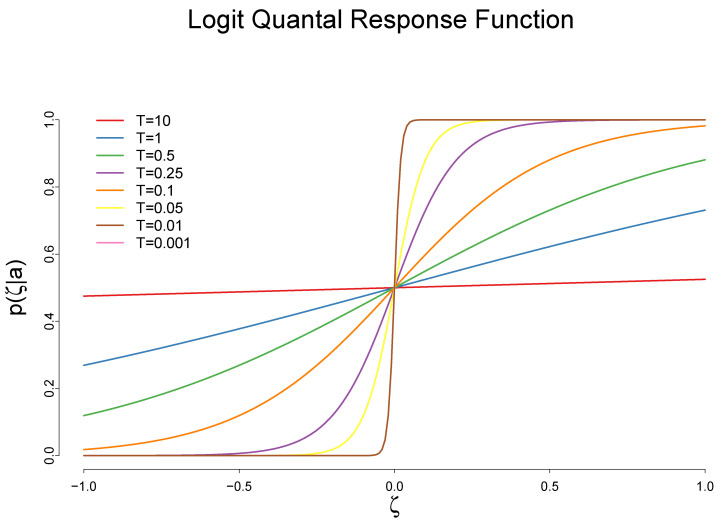
A quantal response function with the behavior temperature *T* and μ=0. The horizontal axis represents the the rate of cost reduction, ζ, while the vertical axis represents the frequency of the adoption of a technology.

Figure 4 shows the quantal response function with the behavior temperature *T*:

This figure shows that the higher the behavior temperature, the more uncertain the decision. Except for the unattainable case when T=0, the function predicts a gradual increase in the frequency of action in response to a higher rate of cost reduction. When *T* is sufficiently large, the quantal response becomes uniform across different actions (the red line). When the behavior temperature *T* is close to zero, the function becomes a step function (the brown line) [14].

### 3.2. The Impact of the Adoption of a New Technology on the Rate of Cost Reduction

The key assumption of the ITC model is that the firm faces a limit in technical progress, the IPF, through which available technologies are constrained by the trade-off between the rates of increase of labor and capital productivity. Under the IPF constraint, an increase in productivity of one input is made possible at the cost of a decrease in productivity of the other input. For example, higher labor productivity growth is coupled with lower capital productivity growth on the innovation possibilities frontier. The trade-off between χ and γ can be represented by the concave function:

(7) $$\gamma =f\left(\chi \right),\;\mathrm{with}\;f^{\prime }<0,\;f^{\prime \prime }<0.$$

**Figure 5:**
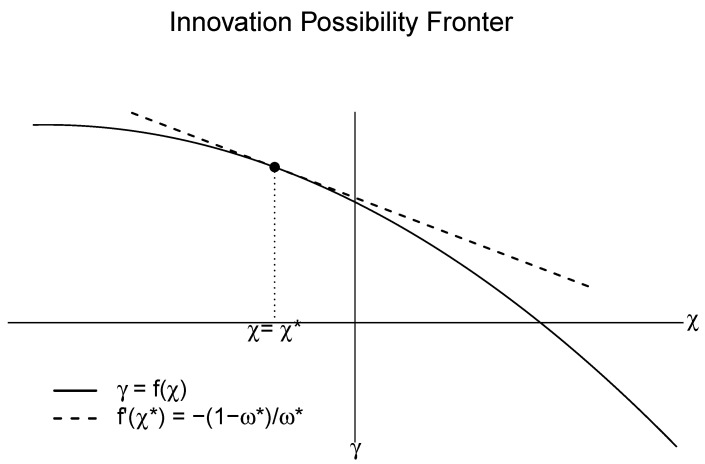
An innovation possibilities frontier. The bold line represents the innovation possibilities frontier (IPF), a functional relationship of the trade-off between χ and γ. The dotted line represents a tangent line of IPF at $\chi =\chi^{*}$. The tangent is the maximum rate of cost reduction given the unit labor and capital cost $\omega =\omega^{*}$ and $\pi =\pi^{*}$.

Figure 5 displays a hypothetical IPF:

The key property of the IPF is that the tangent of the function is the negative factor-price ratio, −π/ω[22]. Depending on a particular π/ω, the firm chooses the corresponding cost-minimizing techniques on the frontier.

The second primary assumption of the QRSE model of technical change is that the adoption of new technology has an impact on the rate of cost reduction through changes in the factor-price ratio. In the model, the typical firm adopts a new technology with a higher than average rate of cost reduction, and therefore, the expected rate of cost reduction conditional on adoption is greater than the expected rate of cost reduction conditional on non-adoption. Now suppose that the act of adopting a new technology does not make any impact on the factor market. In this case, the difference in the conditional expectations of the rate of cost reduction will not be corrected, so that the firm with a new technology will continue to have a higher rate of cost reduction. However, if the new technology changes the factor prices in the market and makes the input initially saved become more expensive, the initial gain in the increased rate of cost reduction will be lost unless the firm finds another new optimal technology for the changed factor-price ratio.

To illustrate, suppose that the firm adopts a labor-saving and capital-consuming technology in response to a higher cost of labor input relative to the capital input. As a result, the firm would require less labor and more capital, which, in turn, will twist the factor-price ratio against the firm’s initial choice by making the labor input cheaper relative to the capital input. Following [15], we can model this negative feedback (or the competitive pressure) in terms of the difference between the two conditional expectation of ζ weighted by the marginal probability of action variable:

(8) $$\begin{array}{c} E\left(\zeta |a\right)p\left(a\right)-E\left(\zeta |\bar{a}\right)p\left(\bar{a}\right)\leq \delta , \end{array}$$

where δ represents the degree of the “ineffectiveness” of the competitive pressure. The larger δ is, the less effective market response is as to the adoption of new technology. When δ=0, it implies that the factor market is so effective that it completely corrects any impacts from the technical change. In contrast, when δ=∞, the cost reduction achieved by a new technology will not be offset by the negative feedback from the adverse changes in the factor-price ratio.

### 3.3. Maximum Entropy Program of the Quantal Response ITC Model

Up to now we have discussed two model assumptions in terms of the interaction between the action variable *A* and the outcome variable ζ. Additionally, we introduce the mean constraint on ζ, E(ζ)=ψ, which represents the typical rate of cost reduction deemed by the cost-minimizing firms in a competitive market. (For a detailed discussion on the first moment constraints in economic models, see [33].) These assumptions can be used as the constraints of our maximum entropy program to find the statistical equilibrium of *A* and ζ. Using the constraints in Equation (7) and (8), and the mean constraint of the model E(ζ)=ψ, the maximum entropy program can be written as follows:

(9) $$\begin{array}{cc} \max & -\int \sum_{a}p\left(A,\zeta \right)\mathrm{Log}\left[p\left(A,\zeta \right)\right]d\zeta ,\\ s.t & \int \sum_{a}p\left(A,\zeta \right)d\zeta =1\\ & \int p(\zeta )\zeta d\zeta =\psi \\ & \int p\left(a,\zeta \right)\zeta d\zeta -\int p\left(\bar{a},\zeta \right)\zeta d\zeta \leq \delta \\ & p\left(A=a|\zeta \right)=\frac{1}{1+e^{-\frac{\zeta -\mu }{T}}}. \end{array}$$

**Figure 6:**
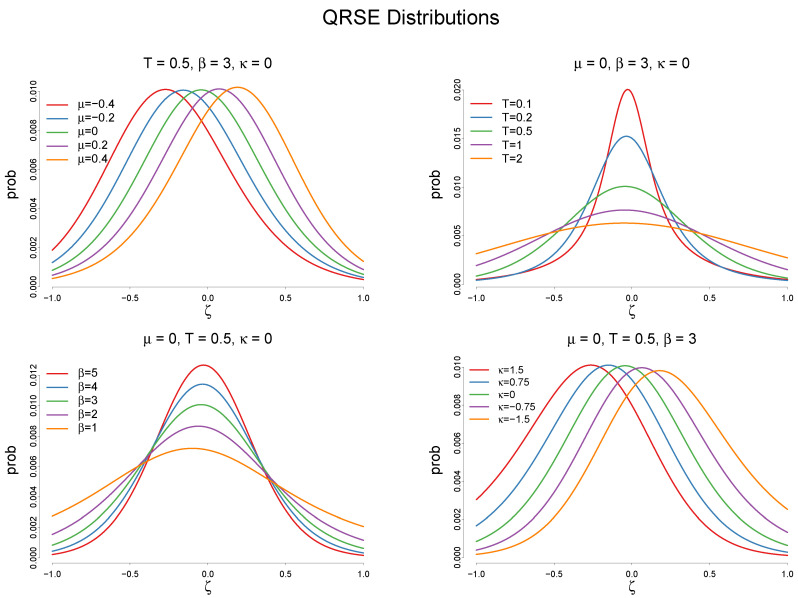
QRSE distributions with different parameter values of μ,T,β, and κ. μ determines the location of the distribution, while κ determines its skewness. κ close to zero represents a symmetric QRSE distribution. Predictably, *T* and β determine how disperse the distribution is. The lower *T* and higher β are, the more spread out the distribution becomes.

The solution to this problem is expressed in terms of the joint distribution $\hat{p}\left(A,\zeta \right)$ which implies two “predicted” marginal distributions, $\hat{p}\left(A\right),\hat{p}(\zeta$), and two conditional distributions, $\hat{p}\left(A|\zeta \right),\hat{p}\left(\zeta |A\right)$. Since the data on the action variable *A* are not observable, we need to express the solution in terms of the marginal distribution of ζ, $\hat{p}\left(\zeta \right)$ to be able to estimate the unknown parameters of the model. The solution to this program then becomes:

(10) $$\begin{array}{c} \hat{p}\left(\zeta \right)=\frac{e^{H_{\mu ,T}}e^{-\beta \mathrm{Tanh}\left[\frac{\zeta -\mu }{2T}\right]\zeta }e^{-\kappa \zeta }}{\sum_{\zeta }e^{H_{\mu ,T}}e^{-\beta \mathrm{Tanh}\left[\frac{\zeta -\mu }{2T}\right]\zeta }e^{-\kappa \zeta }}, \end{array}$$

where $H_{\mu ,T}\left(\zeta \right)=H\left[\frac{1}{e^{-\frac{\zeta -\mu }{T}}+1},\frac{1}{e^{\frac{\zeta -\mu }{T}}+1}\right]$ and Tanh[α] is the hyperbolic tangent function, written as $\frac{e^{2\alpha }-1}{e^{2\alpha }+1}$. (See [15] for the proof. The solution is in discrete terms and requires the coarse-grained bins of ζ.) There are four unknown parameters, μ,T,β and κ. μ and *T* are the location and the shape parameter of the quantal response function, β is the Lagrangian multiplier of the δ constraint and implies the impact of adoption *A* on the cost reduction ζ, and κ is the Lagrangian multiplier of the mean constraint ψ, which determines the skewness of the distribution. A graphical characterization of distribution (10) is provided in Figure 6.

## 4. Bayesian Estimation of the Model

### 4.1. Model Specification

In estimating μ,T,β and κ, we will rely on the Bayesian inference. In setting up the likelihood function, we utilize the observed distribution of ζ and use it as a reference distribution whose divergence from $\hat{p}\left(\zeta \right)$ determines the likelihood of the four unknown parameters. (One could use the derived maximum entropy distribution as a likelihood function and evaluate it directly. This method requires a numerical integration of the partition function since it does not have a closed form solution. One small issue with this method is that the integration needs to be repeated at each evaluation, which can slow down the computation, especially when we sample from it with a large number of iterations and multiple chains in the simulation.) For the comparison of the predicted and the observed distribution, we use the Kullback-Leibler divergence [34]. The KL divergence measures a discrepancy between two distributions and thus has been widely used as a measure of comparing different distributions in the context of many statistical inferences such as model estimation, model selection, and classification. The KL divergence of the discrete distribution of *p* given a known distribution *q*, $D_{\mathrm{KL}}\left(p∥q\right)$, is defined as follows:

(11) $$\begin{array}{ccc} D_{\mathrm{KL}}\left(p∥q\right) & = & \sum_{i}p\left(x_{i}\right)\;\log\frac{p(x_{i})}{q(x_{i})}\\ & = & H(p,q)-H(p). \end{array}$$

Regarding the goodness of fit test, the KL divergence measures how close the distribution of actual data p(x) is to the candidate model of probability distribution q(x). A lower KL divergence implies that it is more likely that the observed distribution of p(x) comes from the presumed model q(x).

Using the KL as a metric, we set up a likelihood function for a lower KL divergence to have a higher likelihood in the function. Any monotonically decreasing function of $D_{\mathrm{KL}}$ can be a candidate. However, the KL divergence itself is actually a good approximation to the log-likelihood for the multinomial model as follows:

(12) $$\begin{array}{c} \log\left[p\left(X|p\right)\right]\approx -n*D_{\mathrm{KL}}\left(X/n∥p\right), \end{array}$$

where $X=(X_{1},X_{2},\ldots ,X_{k})$ is the random variable that represents the number of times the outcome *k* occurs, $p=(p_{1},p_{2},\ldots ,p_{k})$ is the probability of outcome *k*, and *n* is the total number of observations. Since we have the coarse-grained bins of observed distribution of ζ, which can be interpreted as a particular realization of multinomial distribution, we can think of the frequency of each outcome as a sample of a multinomial model with the predicted probability of ζ from Equation (10). Therefore, denoting the coarse-grained bin of ζ as $\bar{p}\left(\zeta \right)$ and remembering that $\hat{p}$ is the maximum entropy distribution, the log-likelihood function becomes:

(13) $$\begin{array}{c} \log\left[p\left(\mu ,T,\beta ,\kappa |\hat{p}\right)\right]=-n*D_{\mathrm{KL}}\left(\bar{p}\left(\zeta \right)|\hat{p}\left(\zeta \right)\right). \end{array}$$

### 4.2. Result

We evaluate $\log[p\left(\mu ,T,\beta ,\kappa |\hat{p}\right)]$ in Equation (14) using a sequence of random samples from the posterior distribution obtained from the Metropolis-Hastings (MH) algorithm, one of Markov Chain Monte Carlo (MCMC) methods. We use 50,000 iterations and 3 chains to recover the marginal posterior distributions of parameters μ,T,β,κ for 7 countries in 8 different years. For all the parameters μ,T,β and κ, we use uninformative priors (uniform priors). For detailed discussions on the MCMC methods, see [35,36,37]. (The result is not greatly sensitive to a wide range of different binning schemes since we have a large number of data points within a relatively small range −100% and −100%.)

#### Parameter Estimation

**Figure 7:**
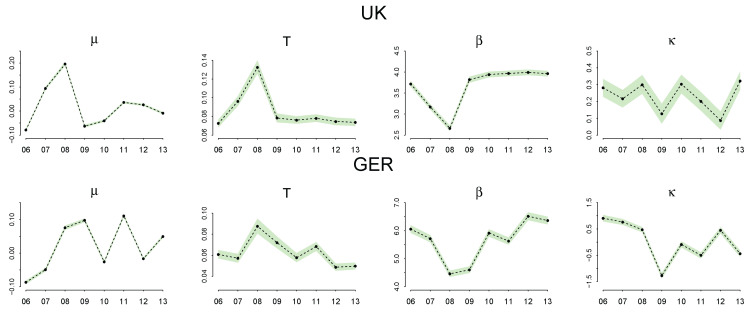

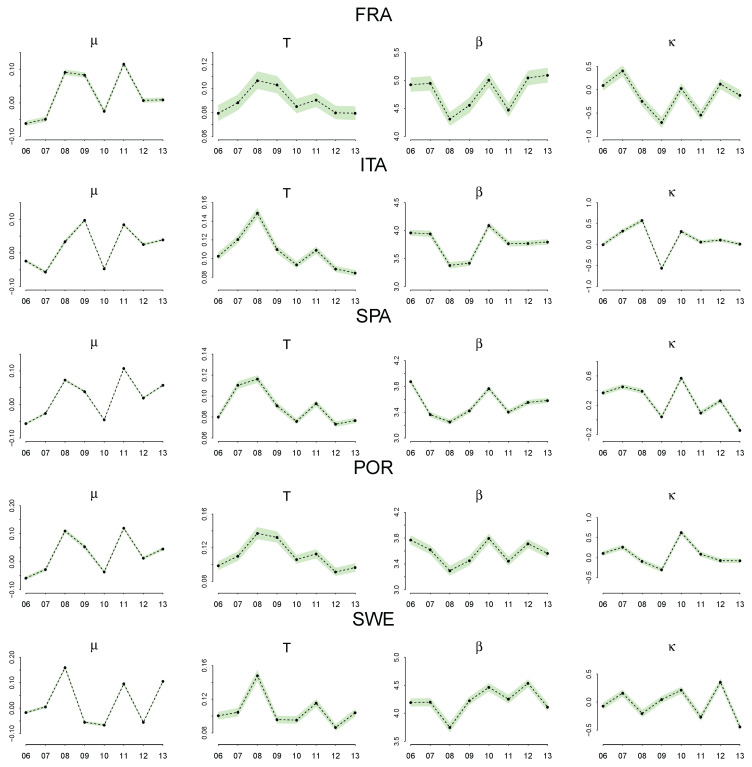
Estimated parameters μ,T,β, and κ along with the 95% credible interval. The year index represents the beginning year from which the growth rate of ζ is calculated. For example, the growth rate in year 2006 is the growth of ζ between 2006–2007.

Figure 7 summarizes the recovered coefficients after 25,000 burn-in periods of the simulation along with 95% credible interval. (All three chains in the simulations are well mixed and properly converge with $\hat{R}$ being 1 in all cases. For a detailed discussion on the convergence diagnosis using $\hat{R}$, see [37].)

The 95% credible interval of the parameters show that the posterior distribution has a very small standard deviation due to the large sample size. An increase in μ and *T* and a decrease in β are noticeable during the 2007–2009 financial crisis. κ is close to zero with no clear patterns. The interpretation of this result is discussed in detail in the following sections.

#### Comparison of Prior and Posterior Distribution: UK 2011

**Figure 8:**
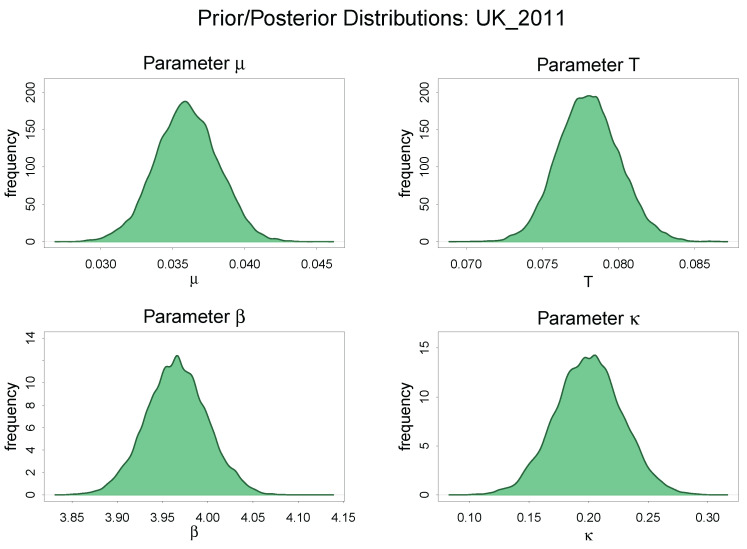
The posterior distributions of μ,T,β and κ.

We can visualize the posterior distributions of the parameters using the estimation from the UK in 2011 as a representative case whose result is more or less the same as other estimations regarding the shape of posterior distribution in Figure 8.

The posterior distributions of all parameters μ,T,β and κ are well recovered and exhibits a unimodal symmetric distribution with a very small standard deviation.

#### Predicted $\tilde{p}\left(\zeta \right)$, $\tilde{p}\left(A|\zeta \right)$, and $\tilde{p}\left(\zeta |A\right)$: UK 2011

**Figure 9:**
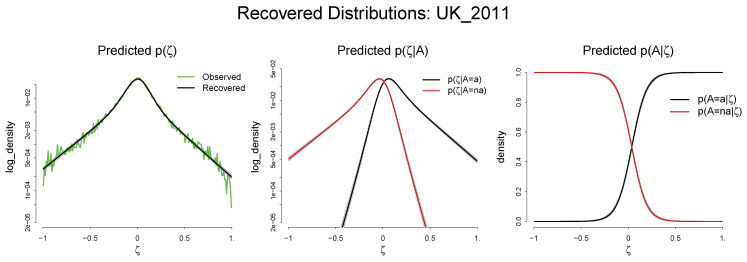
The recovered distribution of $\tilde{p}\left(\zeta \right)$, $\tilde{p}\left(A|\zeta \right)$, and $\tilde{p}\left(\zeta |A\right)$. Distributions are recovered using the mean value of estimated parameters along with 95% credible interval expressed as the scattered gray points.

Using the estimated parameters, we can recover the marginal and the conditional distributions: $\tilde{p}\left(\zeta \right)$, $\tilde{p}\left(A|\zeta \right)$, and $p(\tilde{\zeta }|A)$. Figure 9 visualizes the recovered distribution, again using the estimation of the UK in 2011 as a representative example.

The first panel in Figure 9 represents the recovered marginal distribution of ζ, $\tilde{p}\left(\zeta \right)$, along with the observed one, $\bar{p}\left(\zeta \right)$. The black line is $\tilde{p}\left(\zeta \right)$ recovered from the mean value of estimated parameters, while the green line is $\bar{p}\left(\zeta \right)$. The visual inspection shows that the model satisfactorily recovers the observed distribution. The informational performance of the fitted distribution based on the Soofi ID shows that $\tilde{p}\left(\zeta \right)$ explains 99.6% of the informational content of $\bar{p}\left(\zeta \right)$. (A detailed discussion on the Soofi ID and the informational performance of all the fitted distributions can be found in Appendix.) The second panel shows the recovered conditional distribution of ζ on the adoption decisions, $\tilde{p}\left(\zeta |A\right)$. $\tilde{p}\left(\zeta |A=a\right)$ is skewed to the right while $\tilde{p}\left(\zeta |A=\bar{a}\right)$ is skewed to the left, implying that the former has a higher expected value of ζ. This confirms the initial model assumption that the conditional expectation of ζ on adoption, E(ζ|A=a), is greater than conditional expectation of ζ on non-adoption, $E(\zeta |A=\bar{a})$ constrained by δ. The final panel displays the recovered quantal response function, the conditional distribution of the adoption decisions on ζ, $\tilde{p}\left(A|\zeta \right)$. A substantial degree of uncertainty exists in adopting a new technology as is shown by the fact that the recovered link function is far from the step function.

### 4.3. Discussion of the Estimated Parameters, μ,T,β and κ

The posterior estimation of μ,T,β and κ for seven countries over eight years summarized in Figure 7 exhibits four important patterns.

**Figure 10:**
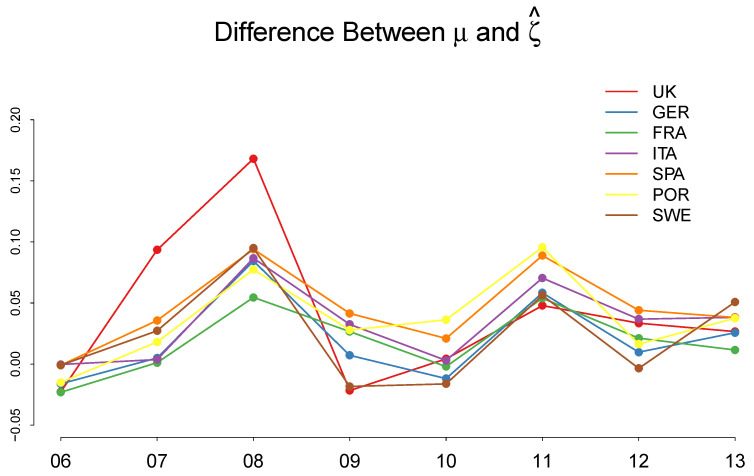
The difference between the recovered μ and the observed mean of $\hat{\zeta }$, $\mu -\hat{\zeta }$.

First, the estimated μ sharply increases during the financial crisis 2007–2009 in all countries. As we discussed before, μ represents the location parameter of the quantal response function that determines the indifference point between adoption and non-adoption. A positive μ means that a positive premium on the potential rate of cost reduction is required for the firm to adopt a new technology with a higher than 50% probability. A sharp increase in μ during the financial crisis demonstrates that the firms became more reluctant to adopt a new technology and needed a higher premium to do so. This point can be more clearly demonstrated by taking the difference between the recovered μ and the observed mean of $\hat{\zeta }$ summarized as follows:

Figure 10 shows that, before the financial crisis in 2006, μ is almost equal to (or slightly smaller than) the average ζ in all countries, implying that the firms did not need an additional premium on the rate of cost reduction above the social average to adopt a new technology. During the financial crisis, however, $\mu -\hat{\zeta }$ sharply increases and turns positive, requiring higher than the average premium for the firms to adopt a new technology with a higher than 50% probability.

Second, the estimated *T* also increases during the crisis period. The parameter *T* is the shape parameter of the quantal response function that determines the spread of the function. The higher *T* is, the more spread out the response function becomes, making the probability of adoption and non-adoption less distinguishable. In economic terms, the spread of the function is interpreted as a degree of the bounded rationality of the economic agent and therefore quantifies how uncertain the economic agent is about her decision. In this regard, an increase in *T* in 2007–2009 can be explained in terms of a higher degree of uncertainty in adopting a new technology during the crisis. This implies that the firms more (or less) frequently adopted those technologies that adversely (or favorably) affected the cost efficiency, incurring more economic losses than other periods.

Third, β decreases during the crisis period. In the maximum entropy program, β is the Lagrangian multiplier of the δ constraint that limits the impact of adoption on the rate of cost reduction. The larger the β is, the smaller the δ, and therefore, the more effective the impact of adoption on the potential rate of cost reductions. The fact that β was lower during the financial crisis suggests that the market effectiveness was lower during the market turmoil than other periods, and therefore, the technological progress of the firms didn’t effectively impact the factor-price on the market.

Fourth, the skewness parameter κ is close to zero and has no clear patterns in most countries, except for Germany and France which have seen a drop in κ during the crisis. In the maximum entropy program, κ is the Lagrangian multiplier of the mean constraint ψ. The fact that κ is close to zero means that the distributions of ζ have been relatively symmetric on the whole.

## 5. Discussion

From the statistical inference perspective, the initial set-up of our model in terms of the joint distribution of ζ and *A* is ill-posed due to the fact that the variable *A* is not observable. The MEP can provide a systematic solution to this problem of limited data [38,39]. The advantage of the MEP method comes from its core inferential logic that the maximum entropy distribution implies the most likely state of the system, regardless of data availability. With adequate economic theories as constraints, the MEP derives the “prior distribution of data”, with some unknown parameters. This predicted distribution can be fitted to the observed data in the way that minimizes the model’s informational loss (the KL divergence). Recovered unknown parameters can be used to infer the unobservable variables, that is, in our case, the adoption decision *A*. This enables us to recover latent distributions involving unobservable variable, such as p(A|ζ) and p(ζ|A), providing a rich picture of the system of economic dynamics.

Another important aspect of our statistical model of technical progress is that it is fundamentally different from the conventional Walrasian equilibrium models. Both models assume that the social outcome is a result of a micro-behavior of the economic agent. However, unlike the “representative agent” in the Walrasian equilibrium model, our model does not assume that the agents are identical. The “typical agent” in our model has a quantal response function with a non-zero behavior temperature as a behavioral rule. Even with the same payoff function, the quantal response makes possible a wide range of heterogenous behaviors. (The statistical model of technical progress is not a single behavioral model but a whole class of different behavioral models. This is because it inherently abstracts from any behavioral assumptions but still predicts the heterogeneity of the economic results due to the non-zero behavior temperature *T* of the quantal response function.) Also, in the Walrasian equilibrium model, the economic agent is inherently atomistic in the sense that she does not make any impact on the aggregate economic outcome. Our model relaxes this assumption and allows a positive degree of interaction so that the typical agent exerts an impact on the aggregate outcome.

## 6. Conclusions

This paper has developed a QRSE model of induced technical change with the binary action variable (adoption or non-adoption of a new technology) and the outcome variable (the rate of cost reduction). Constraints are put on the interactions of the action- and outcome-based ITC model. The impact of cost reduction on the adoption is modeled using the payoff function of the firm, which assumes that higher rates of cost reduction yield higher payoffs with a positive degree of uncertainty in the decision process. The impact of the adoption of new technology on the rate of cost reduction is formulated utilizing the idea of the economic impact of input-saving technical progress on the factor-price ratio.

The principle of maximum entropy is then applied to find the statistical equilibrium of ζ and *A* given four parameters, μ,T,β and κ of the model. The recovered joint distribution of ζ and *A*, which implies the marginal distribution of ζ, is compared to the observed distribution of ζ using the relative entropy, to set up a likelihood function for unknown parameters. Using the uniform prior distribution, the posterior distributions of the parameters are estimated using the MCMC simulation whose 95% credible intervals are used to recover relevant distributions of our interest, that is, the marginal distribution of ζ, p(ζ), and the conditional distributions, p(A|ζ) and p(ζ|A).

Recovered parameters and distributions imply some interesting features in the patterns of firms’ technical progress in the seven advanced OECD countries. During the financial crisis in 2007–2009, the firms became more reluctant to adopt a new technology and needed a higher premium on the potential rate of cost reduction to do so, had a higher degree of uncertainty in adopting a new technology, and the technological progress didn’t effectively impact the factor-price on the market.

## Appendix A. Summary Statistics of the Frequency Distributions of *γ*, *χ*, and *ζ*

The following Table A1 summarizes the mean of γ, χ, $\hat{k}$, and ζ. $\hat{k}$ is defined as γ−χ and represents the change in capital intensity L/K.

**Table A1:** The mean of $\gamma ,\;\chi ,\;\hat{k}$ and ζ of 7 different countries averaged from 2006–2013.

Country	UK	GER	FRA	ITA	SPA	POR	SWE
γ (%)	0.98	0.43	1.15	−0.80	−1.02	0.65	1.10
χ (%)	−6.22	−1.13	−1.89	−3.38	−6.57	−4.43	−2.12
$\hat{k}$ (%)	7.20	1.56	3.04	2.58	5.55	5.08	3.22
ζ (%)	−2.10	−0.08	0.33	−1.49	−2.46	−0.91	−0.24

The summary statistics show that there has been a labor-saving technical change between 2006 and 2013 in all but Italy and Spain, as is demonstrated by the positive γ and the negative χ. The capital intensity has dramatically increased during this period. Except for France, ζ is shown to be negative implying that firms in those countries have, on average, failed to reduce costs. This unfavorable situation is caused by relatively low (or even negative) growth rate of labor productivities along with a fairly large decrease in the growth rate of capital productivity, especially during the financial crisis in 2007–2009 that adversely affected the firm’s revenue.

## Appendix B. Soofi ID Index

Soofi et al. [40] and Soofi and Retzer [41] develop information distinguishability (ID), in which the actual distribution f(x) is compared against the maximum entropy distribution of *x* as the candidate modle $f^{*}\left(x\right)$. The ID index is defined by:

(A1) $$\begin{array}{c} ID\left(f:f^{*}|\theta \right)=1-\exp[-D_{\mathrm{KL}}(f∥f^{*}|\theta )], \end{array}$$

where *f* is the actual data distribution and $f^{*}$ is the maximum entropy distribution of *x* with the corresponding parameters θ. The ID index is a normalized measure so $0\leq ID(f∥f^{*}\left|\theta \right)\leq 1$. As the KL divergence, a smaller value of ID index implies that the actual distribution is similar to the maximum entropy distribution. When $ID(f∥f^{*}\left|\theta \right)\approx 0$, *f* and $f^{*}$ are approximately same Soofi et al. [40]. Finally, different distributions $f_{1}\left(x|\theta \right)$, $f_{2}\left(x|\theta \right)$,…, $f_{n}\left(x|\theta \right)$ are ID distinguishable if $ID(f_{1}∥f^{*}\left|\theta \right)\neq ID(f_{2}∥f^{*}\left|\theta \right)\neq ,\ldots ,\neq ID(f_{n}∥f^{*}\left|\theta \right)$ (Soofi, 1995). The following Table A2 summarizes the Soofi ID index of all the fitted distributions of $\tilde{p}\left(\zeta \right)$

**Table A2:** The Soofi ID index of all the fitted distributions of $\tilde{p}\left[\zeta \right]$ for all 7 countries over 8 years.

Year	UK	GER	FRA	ITA	SPA	POR	SWE
2006	0.006	0.069	0.043	0.006	0.010	0.010	0.006
2007	0.020	0.056	0.060	0.008	0.010	0.007	0.011
2008	0.061	0.061	0.029	0.025	0.010	0.009	0.015
2009	0.007	0.050	0.039	0.020	0.003	0.012	0.008
2010	0.007	0.027	0.035	0.008	0.013	0.018	0.015
2011	0.004	0.014	0.019	0.004	0.003	0.007	0.007
2012	0.008	0.017	0.018	0.003	0.002	0.009	0.004
2013	0.005	0.015	0.027	0.004	0.005	0.008	0.008

## References

[1] Jaynes E.T., Levine R.D., Tribus M. (1978). Where Do We Stand on Maximum Entropy? In The Maximum Entropy Formalism.

[2] Jaynes E.T. (1957). Information theory and statistical mechanics. Phys. Rev..

[3] Farjoun E., Machover M. (1983). Laws of Chaos: Probabilistic Approach to Political Economy.

[4] Foley D.K. (1994). A Statistical Equilibrium Theory of Market. J. Econ. Theory.

[5] Stutzer M.J. (1995). A Bayesian Approach to Diagnostic of Asset Pricing Models. J. Econ..

[6] Stutzer M.J. (2010). Simple Entropic Derivation of a Generalized Black-Scholes Option Pricing Model. Entropy.

[7] Sims C.A. (2003). Implications of rational inattention. J. Monetary Econ..

[8] Smith E., Foley D.K. (2008). Classical thermodynamics and economic general equilibrium theory. J. Econ. Dyn. Control.

[9] Toda A.A. (2010). Existence of a statistical equilibrium for an economy with endogenous offer sets. Econ. Theory.

[10] Toda A.A. (2015). Bayesian general equilibrium. Econ. Theory.

[11] Lux T., Rosser J.B. (2009). Applications of statistical physics in finance and economics. Handbook on Complexity Research.

[12] Zhou R., Cai R., Tong G. (2013). Applications of Entropy in Finance: A Review. Entropy.

[13] Rosser J.B. (2016). Entropy and econophysics. Eur. Phys. J. Spec. Top..

[14] Yang J. (2017). Information theoretic approaches in economics. J. Econ. Surv..

[15] Scharfenaker E., Foley D.K. (2017). Quantal Response Statistical Equilibrium in Economic Interactions: Theory and Estimation. Entropy.

[16] Michl T., Foley D.K. (1999). Growth and Distribution.

[17] Alfarano S., Milaković M. (2008). Does Classical Competition Explain the Statistical Features of Firm Growth?. Econ. Lett..

[18] Alfarano S., Milaković M., Irle A., Kauschke J. (2012). A Statistical Equilibrium Model of Competitive Firms. J. Econ. Dyn. Control.

[19] Jaynes E.T. (2003). Probability Theory: The Logic of Science.

[20] Kapur J.N. (1998). Maximum-Entropy Models in Science and Engineering.

[21] Von Weisäcker C.C. (2010). A New Technical Progress Function (1962). Ger. Econ. Rev..

[22] Kennedy C. (1964). Induced Bias in Innovation and the Theory of Distribution. Econ. J..

[23] Samuelson P.A. (1965). A Theory of Induced Innovation along Kennedy-Weisäcker Lines. Rev. Econ. Stat..

[24] Drandakis E.M., Phelps E.S. (1966). A Model of Induced Invention, Growth and Distribution. Econ. J..

[25] Foley D. (2003). Endogenous Technical Change with Externalities in a Classical Growth model. J. Econ. Behav. Organ..

[26] McFadden D.L. (1976). Quantal Choice Analaysis: A Survey. Ann. Econ. Soc. Meas..

[27] McFadden D.L. (1976). Economic choices. Am. Econ. Rev..

[28] Train K. (2009). Discrete Choice Methods with Simulation.

[29] McKelvey R.D., Palfrey T.R. (1995). Quantal Response Equilibria for Normal Form Games. Games Econ. Behav..

[30] Chen H.C., Friedman J.W., Thisse J.F. (1997). Boundedly Rational Nash Equilibrium: A Probabilistic Choice Approach. Games Econ. Behav..

[31] McKelvey R.D., Palfrey T.R. (1998). Quantal Response Equilibria for Extensive Form Games. Exp. Econ..

[32] Wolpert D., Braha D., Minai A., Bar-Yam Y. (2006). Information Theory—The Bridge Connecting Bounded Rational Game Theory and Statistical Physics. Complex Engineered Systems.

[33] Dos Santos P.L. (2017). The Principle of Social Scaling. Complexity.

[34] Kullback S., Leibler R. (1951). On information and sufficiency. Ann. Math. Stat..

[35] Gamerman D., Lopes H.F. (2008). Markov Chain Monte Carlo: Stochastic Simulation for Bayesian Inference.

[36] Minh D.D.L., Minh D.L. (2015). Understanding the Hastings Algorithm. Commun. Stat. Simul. Comput..

[37] Gelman A., Carlin J.B., Stern H.S., Dunson D.B., Vehtari A., Rubin D.B. (2013). Bayesian Data Analysis.

[38] Golan A. (2002). Information and Entropy Econometrics—Editor’s View. J. Econ..

[39] Golan A., Judge G.G., Miller D. (1996). Maximum Entropy Econometrics: Robust Estimation with Limited Data.

[40] Soofi E.S., Ebrahimi N., Habibullah M. (1995). Information Distinguishability with Application to Analysis of Failure Data. J. Am. Stat. Assoc..

[41] Soofi E., Retzer J. (2002). Information indices: Unification and applications. J. Econ..

